# Potato Virus Y NIb Multifunctional Protein Suppresses Antiviral Defense by Interacting with Several Protein Components of the RNA Silencing Pathway

**DOI:** 10.3390/ijms27031208

**Published:** 2026-01-25

**Authors:** Prakash M. Niraula, Saniyaa Howell, Chase A. Stratton, Michael T. Moore, Matthew B. Dopler, Muhammad I. Abeer, Michael A. Gitcho, Vincent N. Fondong

**Affiliations:** 1 Department of Biological Sciences, Delaware State University, 1200 North DuPont Highway, Dover, DE 19901, USAcstratton@desu.edu (C.A.S.); mmoore@desu.edu (M.T.M.); mgitcho@desu.edu (M.A.G.); 2 DNA and Data Science Core (dsDNA), Delaware State University, 1200 North DuPont Highway, Dover, DE 19901, USA; 3 3UT Health San Antonio, 7703 Floyd Curl Drive, San Antonio, TX 78229, USA

**Keywords:** AGO4, HSP70, HSP90, nuclear inclusion protein b (NIb), RdRP, SGS3, siRNA, suppressor of RNA silencing

## Abstract

*Potyvirus* genomes are expressed as a single large open reading frame, which is translated into a polyprotein that is post-translationally cleaved by three virus-encoded proteases into 10 functional proteins. Several of these potyviral proteins, including nuclear inclusion protein b (NIb), are multifunctional. Here, using the classic GFP silencing in *Nicotiana benthamiana* gfp-transgenic plants, we show that potato virus Y (PVY) NIb, in addition to its canonical role as the viral RNA-dependent RNA polymerase (RdRP), functions as a suppressor of RNA silencing. Mutational analyses reveal a previously unreported NIb nuclear localization signal (NLS) consisting of a triple-lysine motif. NIb suppression of RNA silencing activity was lost when the NLS was mutated, suggesting that nuclear localization is required for NIb suppression of RNA silencing activity. Analysis of sequenced GFP siRNAs revealed three reproducible hotspot regions at ≈175 nt, ≈320–330 nt, and a broader 3′-proximal region spanning ≈560–700 nt that contains multiple local maxima. These data show differences in the positional distribution of siRNAs between samples expressing NIb and those expressing NIb^Del3×2^, the NIb null mutant that does not suppress RNA silencing. However, the positional distribution of GFP-derived small RNAs across the transgene differed modestly between NIb and NIb^Del3×2^, while both treatments showed the same three reproducible hotspot regions. Furthermore, NIb was found to interact with four key RNA silencing pathway proteins—AGO4, HSP70, HSP90, and SGS3. Except for HSP90, each of these proteins showed degradation products that were absent in NIb mutants that did not suppress RNA silencing. These findings support a role for NIb in countering host defense during virus infection.

## 1. Introduction

Diseases caused by potato virus Y (PVY) are a serious challenge facing potato (*Solanum tuberosum* L.) production worldwide. To date, at least nine recombination patterns of PVY^O^ and PVY^N^ sequences have been identified in potato-infecting PVY isolates, the three most common recombinant patterns being found in strains PVY^NTN^, PVY^N:O^, and PVY^N-Wi^ [1,2]. PVY has a positive single-stranded RNA [(+)ssRNA)] genome that has a single open reading frame (ORF), which undergoes proteolytic processing upon translation, producing 10 functional proteins: P1, HcPro, P3, 6K1, CI, 6K2, VPg, NIa, NIb, and CP [3]. Proteolytic processing is carried out by three virus-encoded proteins, proteinases P1 and HcPro, and the NIa protease [4,5,6]. During replication, an additional polyprotein, P3N-PIPO, is produced due to RdRP slippage, resulting in frameshift mutation at the P3 cistron [7,8,9].

The catalytic subunit of the NIb (nuclear inclusion protein b) functions as an RNA-dependent RNA polymerase (RdRP) and is therefore responsible for viral genome replication and mRNA synthesis. During genome replication, NIb localizes to endoplasmic reticulum (ER) membranes by interacting with the 6K2–VPg–NIaPro polyprotein, leading to the assembly of the viral replication complex (VRC), where replication takes place [10,11,12]. NIb has equally been found to recruit important VRC components, including poly(A)-binding protein (PABP) [13], PABP2, PABP8, eukaryotic elongation factor 1A (eEF1A), and heat shock cognate protein (HSC70) [12,14,15,16]. The polymerase activity of NIb is regulated by the autophagy protein, Beclin1 (or ATG6), which binds NIb at its conserved GDD motif and targets NIb for autophagic degradation [17].

NIb engages in multiple immune response interactions; for instance, it can suppress NPR1-mediated immune signaling and activate effector-triggered immunity [10,17,18,19,20]. Due to this broad interactome, NIb has been dubbed the potyvirus ‘most sticky’ protein [12]. Most RNA viruses, including potyviruses, replicate in the cytoplasm. However, the NIb protein contains nuclear localization signals (NLSs) and is found in both the cytoplasm and nucleus, where together with NIa, it forms amorphous or crystalline nuclear inclusions (NIs) in infected cells [12,21,22,23]. Hence, nuclear localization likely enables post-translational modification, including sumoylation [18], and provides a favorable cellular environment for interaction with host proteins.

Plants use the RNA silencing mechanism to counter invading viruses. To establish a successful infection, therefore, the virus must overcome this host antiviral defense. Viruses achieve this by encoding suppressors of RNA silencing, and some viruses have been shown to encode more than one suppressor of RNA silencing, including East African cassava mosaic Cameroon virus (EACMCV), which encodes three suppressors, namely AC4, AV2, and TrAP [24,25,26]; citrus tristeza virus, which encodes p20 p23, and CP [27]; and beet necrotic yellow vein virus, which encodes p14 and p31 [28,29]. To date, three potyviral silencing suppressors have been reported, including the extensively characterized HcPro [30,31,32,33,34,35,36,37], turnip mosaic virus (TuMV) VPg [38], and cucumber vein yellowing ipomovirus P1 serine protease [39]. Here, we show that PVY NIb suppresses RNA silencing and that this function is dependent on NIb nuclear localization. Accordingly, we identify the motif ^135^KKK^137^ as a previously unreported PVY NIb NLS and show that deletion of the terminal 18 amino acids inhibits NIb nuclear localization. We also show that NIb suppression of RNA silencing is accounted for by its interaction with several protein components of the RNA silencing pathway.

## 2. Results

### 2.1. The PVY NIb Is a Suppressor of RNA Silencing

**Figure 1:**
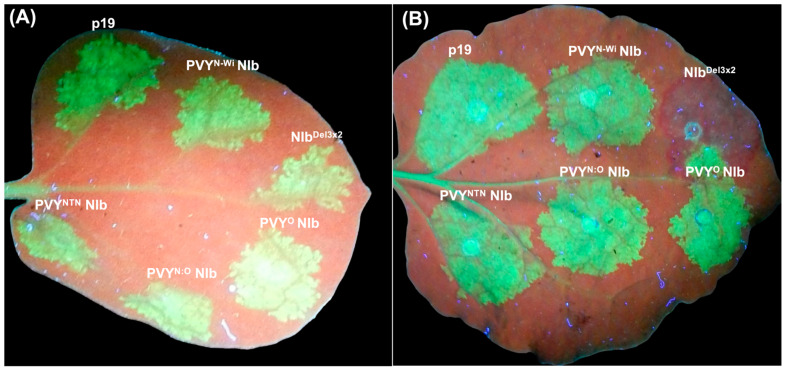
PVY NIb suppresses GFP silencing in *N. benthamiana* 16c gfp-transgenic plants. Leaves were co-infiltrated with 35s::GFP and wild-type NIb constructs or tomato bushy stunt virus p19, then imaged under UV light at (**A**) 2- and at (**B**) 10 days post-infiltration (dpi). Patches co-infiltrated with 35s::GFP and NIb of PVY^NTN^, PVY^O^, PVY^N:O^, and PVY^N-Wi^, and patches containing p19 continued to display GFP green fluorescence. In contrast, the patch co-infiltrated with 35s::GFP and NIb null mutant, NIb^Del3×2^, had lost green fluorescence and showed red chlorophyll autofluorescence, indicating GFP silencing.

The potyvirus NIb is a multifunctional protein with distinct functional domains [7,12,40]. To test whether NIb suppresses host antiviral defense, we used the classic *N. benthamiana* line 16c plant model [41]. Line 16c plants constitutively express the GFP transgene and enable testing of putative viral suppressors of gene silencing. NIb cistrons from four PVY strains (PVY^NTN^, PVY^O^, PVY^N:O^, and PVY^N-Wi^) were each cloned under the control of cauliflower mosaic virus (CaMV) 35S promoter in pEarleyGate101 binary vector [42]. Tomato bushy stunt virus p19 silencing suppressor was used as the RNA silencing suppressor positive control. For the negative control, we generated a null mutant of PVY^NTN^ NIb, NIb^Del3×2^. These constructs were introduced to *Agrobacterium* tumefaciens strain GV3101 and used for co-infiltration with 35s::GFP into *N. benthamiana* 16c plants as described previously [26]. GFP fluorescence in infiltrated leaves was monitored using a handheld longwave UV lamp. By 2 days post-inoculation (dpi), all infiltrated leaf patches displayed intense green fluorescence (Figure 1A). However, by 7 dpi, the level of green fluorescence had started declining and was replaced by red autofluorescence from chlorophyll molecules in patches co-infiltrated with NIb^Del3×2^/35S::GFP, while leaf patches co-infiltrated with each NIb/35s::GFP continued to display intense green fluorescence. At 10 dpi, tissues co-infiltrated with NIb^Del3×2^/35S::GFP displayed intense red fluorescence while the wild-type tissues continued to display intense green fluorescence (Figure 1B). Therefore, in NIb-expressing tissues, functional NIb maintains GFP expression by countering *gfp* silencing, while silencing is established in tissues expressing the NIb^Del3×2^ null mutant.

### 2.2. Characterization of NIb Suppression of RNA Silencing

**Figure 2:**
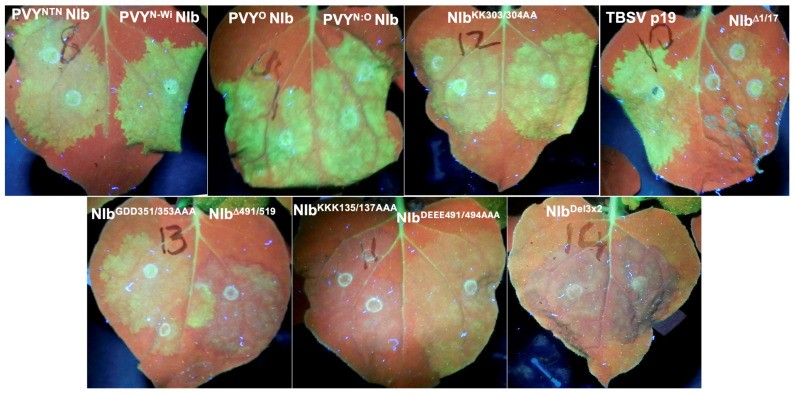
NIb domains and motifs required for RNA silencing suppression. *N. benthamiana* 16c plant leaves were co-infiltrated with 35s::GFP and NIb from four PVY strains (PVY^NTN^, PVY^O^, PVY^N:O^, PVY^N-Wi^), as well as with p19, and seven PVY^NTN^ NIb mutants (NIb^Δ1/17^, NIb^KKK135/137AAA^, NIb^KK303/304AA^, NIb^DEEE491/494AAA^, NIb^GDD351/353AAA^, NIb^Δ4491/519^, and NIb^Del3×2^), respectively. By 10 dpi, NIb^Δ1/17^, NIb^KKK135/137AAA^, NIb^DEEE491/494AAA^, NIb^Δ4491/519^, and NIb^Del3×2^ had lost much of the GFP green fluorescence. In contrast, NIb and NIb^KK303/304AA^ continued to display green fluorescence, while NIb^GDD351/353AAA^ showed only weak green fluorescence.

To characterize NIb suppression of RNA silencing, we produced mutations of PVY^NTN^ NIb, including NIb^Δ1/17^, NIb^Δ491/519^, NIb^KKK135/137AAA^, NIb^KK303/304AA^, NIb^GDD351/353AAA^, NIb^DEEE491/494AAA^, and NIB^Del3×2^ as described in Materials and Methods. These constructs and wild-type NIb cistrons were each co-infiltrated with 35s::GFP into *N. benthamiana* line 16c plant leaves. Examination of co-infiltrated leaf patches showed that at 2 dpi, all co-infiltrated tissues displayed green fluorescence, similar to Figure 1A. However, by 10 dpi, tissues expressing NIb^DEEE491/494AAA^, NIb^GDD351/353AAA^, NIb^Δ1/17^, NIb^KKK135/137AAA^, NIb^Δ491/519^, and NIb^Del3×2^ had lost much of the green fluorescence, which was progressively replaced by red chlorophyll fluorescence, while tissues expressing NIb^KK303/304AA^ and the wild-type cistrons continued to display green fluorescence (Figure 2).

**Figure 3:**
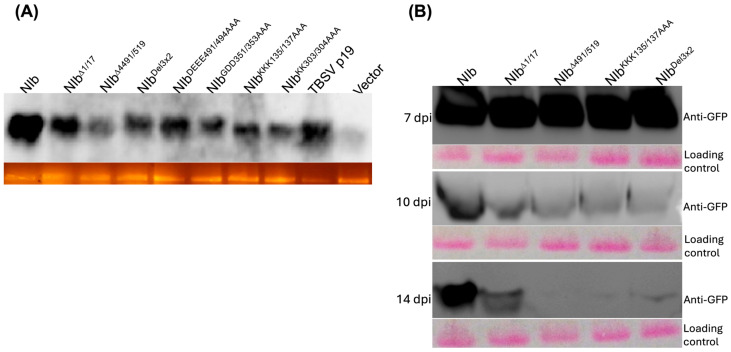
NIb suppresses RNA silencing. (**A**) Northern blot detection of GFP RNA at 7 dpi. Total RNA was isolated from *N. benthamiana* line 16c plant leaves co-infiltrated with 35s::GFP and each of the following: NIb, NIb^Δ1/17^, NIb^Δ4491/519^, NIb^Del3×2^, NIb^DEEE491/494AAA^, NIb^GDD351/353AAA^, NIb^KKK135/137AAA^, NIb^KK303/304AA^, TBSV p19, and an empty vector, and then probed with a biotin-labeled GFP probe. Very low levels of GFP transcripts were observed in tissues co-infiltrated with an empty vector and GFP, while high levels of GFP transcripts were observed in tissues containing NIb and p19. (**B**) Western blot analysis of GFP protein in infiltrated 16c leaves at 7, 10, and 14 dpi. At 7 dpi, leaves co-expressing GFP with wild-type NIb showed high levels of GFP protein compared to much lower GFP levels in leaves expressing NIb mutants NIb^Δ1/17^, NIb^KKK135/137AAA^, NIb^Δ4491/519^, and NIb^Del3×2^. By 10 dpi, except for NIb^Δ1/17^, GFP was barely detectable in NIb mutant samples, and by 14 dpi, GFP was almost undetectable in NIb^Δ4491/519^, NIb^Del3×2^, and NIb^KKK135/137AAA^ samples, while leaves expressing wild-type NIb maintained high GFP protein accumulation.

The fact that at 2 dpi, there were similar levels of GFP fluorescence in all constructs tested indicates that there were similar GFP expression levels. To confirm that loss of GFP fluorescence over time was due to gene silencing, we carried out a Northern blot analysis of GFP transcripts at 7 dpi. Results generally reflect the loss of GFP fluorescence; for example, very low levels of GFP transcripts were observed in tissues co-infiltrated with an empty vector and 35s::GFP, while very high levels of GFP transcripts were observed in tissues containing PVY^NTN^ NIb and p19 (Figure 3A).

We further compared GFP levels in a Western blot analysis using anti-GFP antibodies over time. At 7 dpi, the levels of GFP were similar in all treatments (Figure 3B), however, at 10 dpi, except for NIb^Δ1/17^, GFP was barely detectable in samples expressing NIb mutants, while leaves expressing wild-type NIb maintained strong GFP protein levels through 14 dpi. These findings confirm that loss of green fluorescence in tissues co-infiltrated with NIb mutants and 35s::GFP was due to PTGS, and that NIb suppresses PTGS.

### 2.3. Nuclear Localization Is Required for NIb Suppression of RNA Silencing

**Figure 4:**
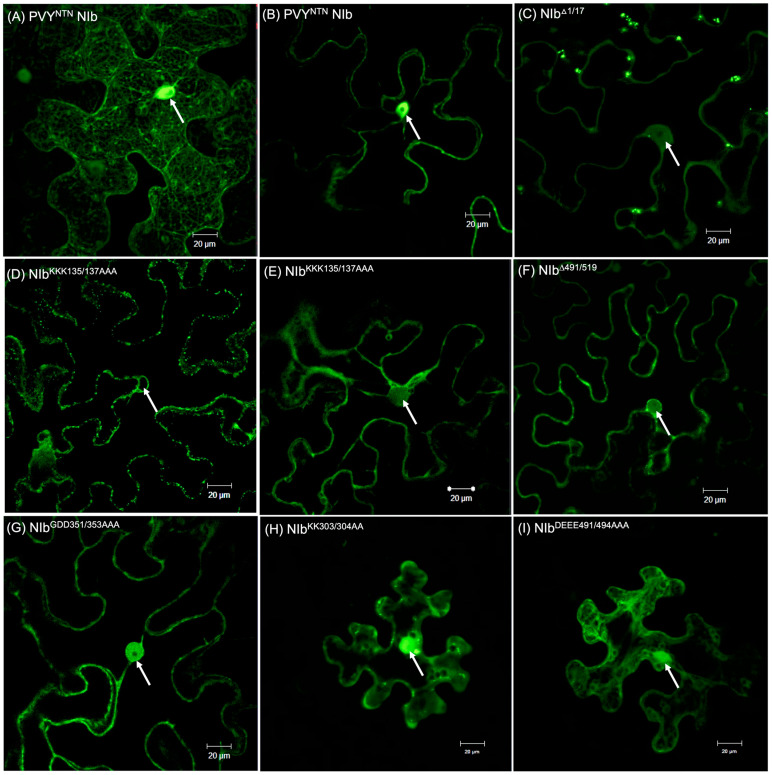
Subcellular localization of NIb and mutants using confocal microscopy. z-stack imaging shows that in the cytoplasm, NIb localizes in cytoplasmic membranes (**A**). Wild-type NIb (**B**), NIb^DEEE491/494AAA^ (**I**), NIb^KK303/304AA^ (**H**), and NIb^GDD351/353AAA^ (**G**) localized to both the nucleus (indicated by arrows) and the cytoplasm. In contrast, NIb^Δ1/17^ (**C**), NIb^KKK135/137AAA^ (**D**) and ((**E**), z-stack), as well as NIb^Δ491/519^ (**F**) localized in the cytoplasm and nuclear membrane, with no detectable nuclear localization. Images were acquired on an upright Zeiss LSM 780 laser-scanning microscope (using a Zeiss 40× C-Apochromat (NA 1.2) objective lens (Carl Zeiss, Inc., Oberkochen, Germany). Additional confocal images of NIb^KKK135/137AAA^ subcellular localization are in Supplementary Figure S1.

Subcellular localization is critical for protein function, as it determines its access to interaction partners and provides the cellular environment needed for interaction. Therefore, to determine whether subcellular localization influences NIb RNA silencing suppression, we investigated the NIb constructs described above, each containing a C-terminal-fused YFP in the pEarleygate101 plasmid. *N. benthamiana* plant leaves were agroinfiltrated with these constructs and examined with a confocal microscope within 48 h of infiltration. Wild-type NIb was observed to localize in the nucleus and the cytoplasm, where it was found to be associated with cytoplasmic membranes where virus replication occurs, consistent with its RdRP role (Figure 4A). Mutants NIb^DEEE491/494AAA^, NIb^KK303/304AA^, and NIb^GDD351/353AAA^ also localized in the nucleus and cytoplasm. In contrast, NIb^Δ1/17^, which was shown to contain a NLS [12], NIb^Δ491/519^, and NIb^KKK135/137AAA^ localized only in the cytoplasm (Figure 4) (Supplementary Figure S1). This suggests that the ^135^KKK^137^ motif is in a previously unreported NIb NLS. The findings also indicate that nuclear localization is required for NIb suppression of RNA silencing. Additionally, the NIb C-terminal 18 amino acid truncation mutant, NIb^Δ491/519^, which does not suppress RNA silencing, was found to not localize in the nucleus, suggesting that the C-terminal truncation contains another NLS or regulates NIb nuclear import.

### 2.4. Small RNA Analysis

**Figure 5:**
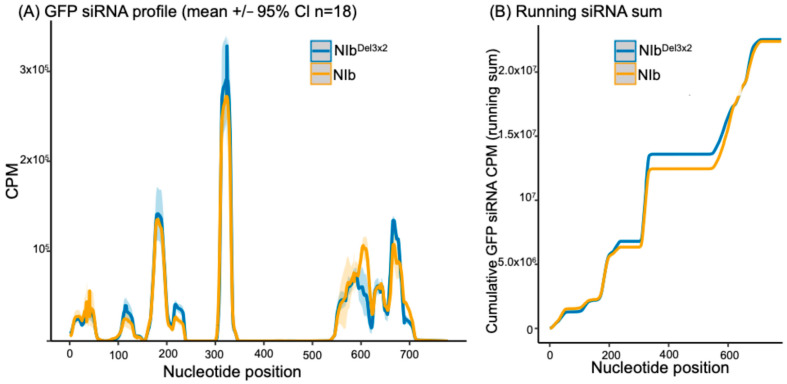
Distribution and aggregate abundance of GFP-derived small RNAs. (**A**) Per-nucleotide coverage of small RNAs mapping to GFP in 16c leaves at 14 dpi (CPM; linear scale). Solid lines show the group mean; ribbons are 95% CIs (*n* = 3 biological replicates per treatment; 6 libraries total). Three reproducible hotspot regions occur at ≈175 nt, ≈320–330 nt, and a broader 3′-proximal region spanning ≈560–700 nt that contains multiple local maxima. Peak heights differ modestly between treatments, indicating positional redistribution rather than uniform gain/loss. (**B**) Cumulative (running) sum of CPM along GFP (CPM normalized per million mapped reads). Absolute GFP-siRNA abundance normalized to total small RNA reads (RPM) is provided in Table S1.

We profiled small RNAs mapped to GFP in 16c leaves co-infiltrated with PVY^NTN^ NIb/35s::GFP, as well as with NIb^Del3×2^/35s::GFP at 14 dpi (*n* = 6 libraries; 3 biological replicates per treatment). Position-wise coverage in counts per million (CPM) on a linear scale showed three reproducible siRNA hotspots across the GFP sequence at ap ≈175 nt, ≈320–330 nt, and ≈560–700 nt (Figure 5A). Peak heights differed modestly between treatments: the 5′-proximal hotspots (≈175 and ≈320–330 nt) tended to be somewhat higher in samples expressing NIb^Del3×2^. Within the broader 3′ hotspot region (≈560–700 nt), the two treatments were broadly similar overall, with a prominent local maximum near ≈675 nt higher in NIb^Del3×2^. This pattern indicates a modest shift, where GFP siRNAs accumulate along the transcript rather than a uniform gain or loss at all sites. We therefore interpret the NIb versus NIb^Del3×2^ contrast as a positional redistribution of GFP-derived siRNAs, rather than a pronounced change in any single peak.

**Figure 6:**
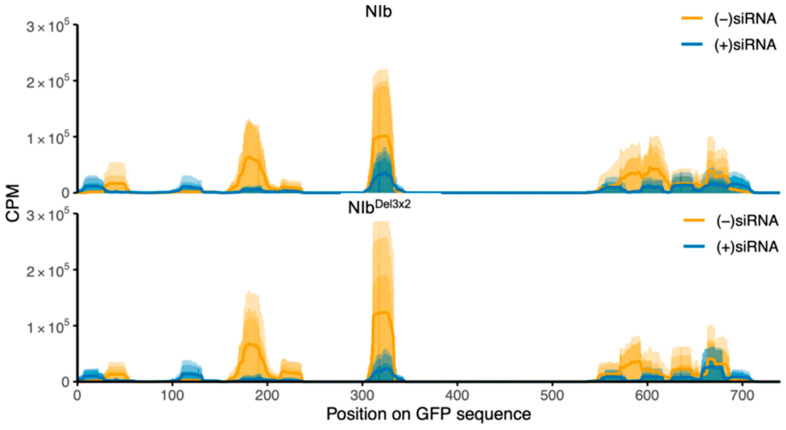
Strand bias of GFP-derived siRNAs. Sense (+) and antisense (−)siRNA coverage along the GFP sequence in line 16c leaves at 14 dpi. Panels show NIb (**top**) and NIb^Del3×2^ (**bottom**) samples separately. Colors indicate strand polarity [(+)siRNA vs. (−)siRNA]. In both treatments, GFP siRNAs are strongly antisense-biased, with (−)siRNAs greatly outnumbering (+)siRNAs.

To assess the aggregate signal in a way that is comparable to the per-nucleotide plots, we integrated CPM along the transcript as a cumulative running sum (Figure 5B). Because CPM values were generated by bamCoverage using normalization per million mapped reads, the cumulative endpoint primarily reflects normalized coverage rather than absolute GFP-siRNA abundance per library. Therefore, to address absolute abundance normalized to sequencing depth, we report total GFP-mapping reads per million total small RNA reads (RPM) for each library, with per-treatment summaries provided in Table S1. GFP-mapping RPM values were higher in NIb^Del3×2^ than NIb (median 4527 vs. 1298 RPM across the six libraries; Wilcoxon rank-sum *p* = 0.10). Consistent with previous studies of the 16c GFP transgene, GFP-mapping siRNAs in both treatments were strongly antisense-biased [43] (Figure 6). In this system, abundant antisense siRNAs are expected when a constitutively expressed transgene becomes a target of RNA silencing because Argonaute-containing RISCs typically retain one strand from each Dicer-generated siRNA duplex as the guide strand (which is complementary to the target RNA), while the opposite passenger strand is discarded. Guide-strand selection depends on properties such as duplex end stability and AGO sorting preferences.

**Figure 7:**
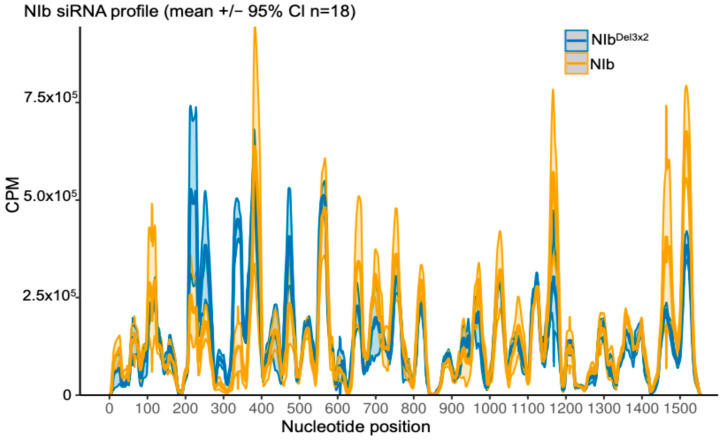
Profiles of small RNAs mapping to the NIb transcript in GFP line 16c plants co-infiltrated with wild-type NIb vs. NIb^Del3×2^ at 14 dpi. Lines indicate mean NIb-siRNA coverage along the NIb coding sequence (reads per million, 1-nt bins; shaded 95% CI, *n* = 3 biological replicates per treatment; 6 libraries total). Overall, NIb-derived siRNAs were present at relatively low levels (note the much lower coverage compared to GFP siRNAs in Figure 5). Notably, leaves expressing wild-type NIb produced slightly more NIb-derived siRNAs than those expressing NIb^Del3×2^. The NIb and NIb^Del3×2^ samples showed broadly similar siRNA distribution profiles along the length of the NIb sequence, suggesting that NIb’s suppressor activity does not eliminate processing of its own transcripts.

**Figure 8:**
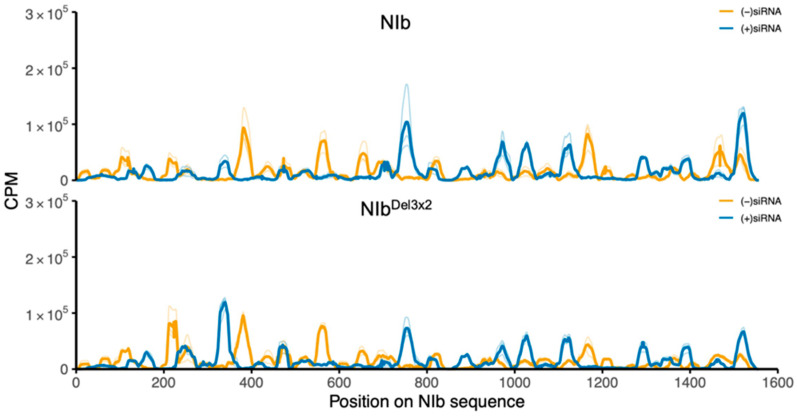
Strand profiles of NIb-derived siRNAs. Sense (+) and antisense (−)siRNA coverage along the NIb coding sequence in *N. benthamiana* line 16c leaves at 14 dpi. Panels show NIb and NIb^Del3×2^ samples separately; colors indicate strand polarity [(+)siRNA vs. (−)siRNA]. In contrast to the GFP-derived siRNA pattern (Figure 7), NIb-derived siRNAs do not show a strong antisense excess: wild-type NIb samples display a modest surplus of (+)siRNAs over (−)siRNAs, whereas NIb^Del3×2^ samples exhibit a more balanced sense/antisense profile.

We also quantified siRNA mapping to the transiently expressed NIb transcripts. NIb-derived siRNAs were readily detectable and broadly distributed across the NIb coding sequence (Figure 7). In contrast to GFP, strand polarity for NIb was not strongly antisense-dominant: NIb samples showed a modest excess of sense-strand siRNAs, whereas NIb^Del3×2^ samples had a more balanced sense/antisense profile (Figure 8). NIb and NIb^Del3×2^ displayed broadly similar positional patterns along the NIb transcript, with only modest differences in local peak heights. Therefore, while NIb does not prevent production of siRNAs from its own transcript, the strand composition of these siRNAs differs from that of GFP-derived species, consistent with differences in how the two RNAs enter the RNA silencing pathway (transgene-derived vs. transiently expressed coding transcript).

### 2.5. NIb Forms a Complex with Four RNA Silencing Pathway Proteins

**Figure 9:**
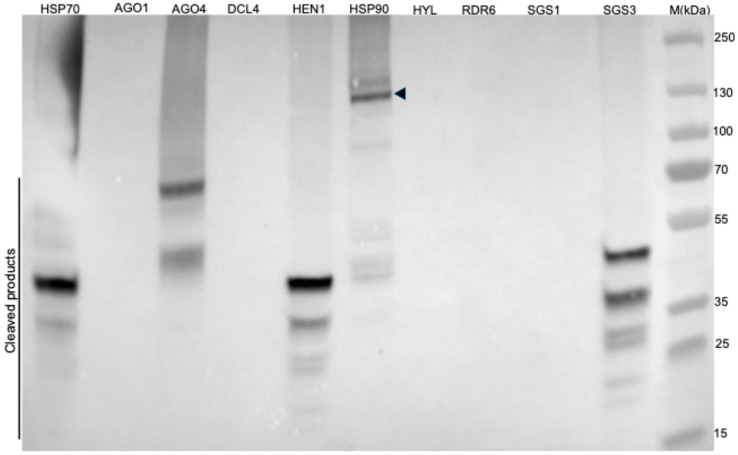
In planta interaction between NIb and host RNA silencing proteins. NIb–HA was co-expressed in *N. benthamiana* leaves with each of the AGO1, AGO4, DCL4, HSP70, HSP90, HYL1, RDR6, SGS1, and SGS3 proteins tagged with 6×HIS. At 5 dpi, total protein was extracted and subjected to Ni–NTA affinity purification of protein complexes, followed by immunoblotting using anti-HA antibodies. NIb was found to co-purify with AGO4 (102 kDa), HSP70 (70 kDa), HSP90 (90 kDa), and SGS3 (72 kDa). No NIb signal was detected in samples with the other candidate proteins, indicating no stable interaction. Samples containing AGO4, HSP70, and SGS3 showed degraded protein products; the arrowhead shows the HSP90/NIb complex. The SDS-PAGE Coomassie blue stain of the total protein lysate for Ni–NTA affinity purification can be found in Supplementary Figure S2. M: PageRuler™ Plus Prestained Protein Ladder, 10 to 250 kDa.

To identify host protein(s) in the RNA silencing pathway that likely interact with NIb, leading to suppression of RNA silencing, we used an in planta protein–protein interaction assay. The pEarleygate101 plasmid used to express NIb, as described above, contains YFP and HA-tag as the C-terminal fusion, and the nine host genes investigated for interaction with NIb contain a C-terminal 6×HIS tag, as described in Materials and Methods. This allowed us to detect interacting complexes using HIS-tag purification beads, followed by immunoblotting with HA-tag antibodies. NIb-HA fusion was therefore co-agroinfiltrated into *N. benthamiana* plants with each host protein. At 5 dpi, infiltrated leaves were collected, and protein complexes were purified using a Ni-NTA (nickel-nitrilotriacetic acid) magnetic bead kit (Thermo Fisher Scientific, Waltham, MA, USA) under native conditions. After native PAGE gel electrophoresis separation of protein complexes, the presence of NIb-HA fusions was tested using HA antibodies in a Western blot assay. Of the nine co-infiltrations, protein bands were observed in AGO4, HSP70, HSP90, and SGS3 samples, indicating interaction between these proteins and NIb (Figure 9). These proteins play key roles in RNA silencing. For example, AGO4 is a key effector of the RNA-directed DNA methylation antiviral pathway [44]; HSP70 and HSP90 are molecular chaperones required for loading siRNAs into RISC [45]; and SGS3 works with RDR6 to convert ssRNAs into dsRNA, which DCL proteins process into secondary siRNA [46]. Therefore, these results suggest that NIb likely suppresses RNA silencing by interacting with at least one of these proteins. Interestingly, NIb/HSP70, NIb/AGO4, and NSP/SGS3 complexes showed smaller molecular weight proteins, but no bands corresponding to the native proteins, possibly indicating that interaction between NIb and these RNA silencing proteins leads to degradation of the interacting complex.

### 2.6. NIb Mutants Deficient in RNA Silencing Suppression Do Not Interact with AGO4, HSP70, or SGS3

**Figure 10:**
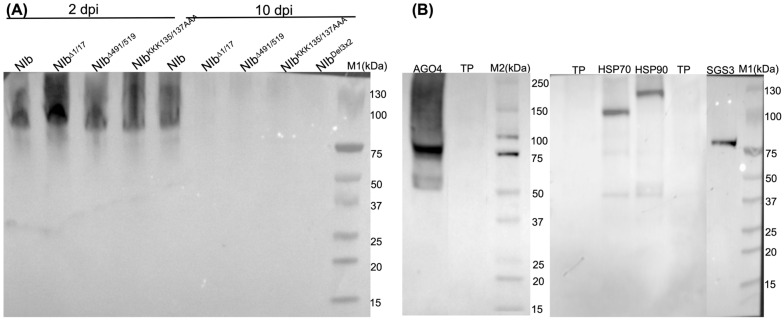
Expression of PVY^NTN^ NIb, NIb^Δ1/17^, NIb^Δ491/519^, NIb^KKK135/137AAA^, and NIb^Δ491/519^, and of AGO4, HSP70, HSP90, and SGS3. (**A**) Total plant proteins were extracted from infiltrated *N. benthamiana* leaves and analyzed by Western blot using anti-HA tag antibodies at 2 dpi and at 10 dpi, when only NIb was detectable. (**B**) To analyze RNA silencing pathway proteins, total protein was first isolated with the Ni-NTA magnetic bead kit prior to detection with anti-HIS-tag antibodies under native conditions. TP: total protein from control plants inoculated with an empty vector; M1: PageRuler™ Plus Prestained Protein Ladder; M2: precision plus protein standard kaleidoscope.

To identify the specific partner(s) with which NIb interacts to suppress RNA silencing, we reasoned that such a protein would not interact with NIb^Δ1/17^, NIb^KKK135/137AAA^, NIb^Δ491/519^, or NIb^Del3×2^, all of which do not suppress RNA silencing. First, NIb constructs, as well as AGO4, HSP70, HSP90, and SGS3, were assessed for expression. Interestingly, whereas NIb^Δ1/17^, NIb^Δ491/519^, and NIb^KKK135/137AAA^ were detectable from total plant protein at 2 dpi, only NIb could be detected at 10 dpi (Figure 10A). This result is consistent with our observation from siRNA analysis (Figure 8) that transiently expressed NIb is a target of RNA silencing. Therefore, detection of NIb at 10 dpi is due to its suppression of RNA silencing. Regarding the RNA silencing proteins, unlike NIb, AGO4, HSP70, HSP90, and SGS3 were only detectable when isolated with the Ni-NTA magnetic bead kit (Figure 10B). HSP70 and HSP90 were observed as dimers given that this analysis was conducted under native conditions.

**Figure 11:**
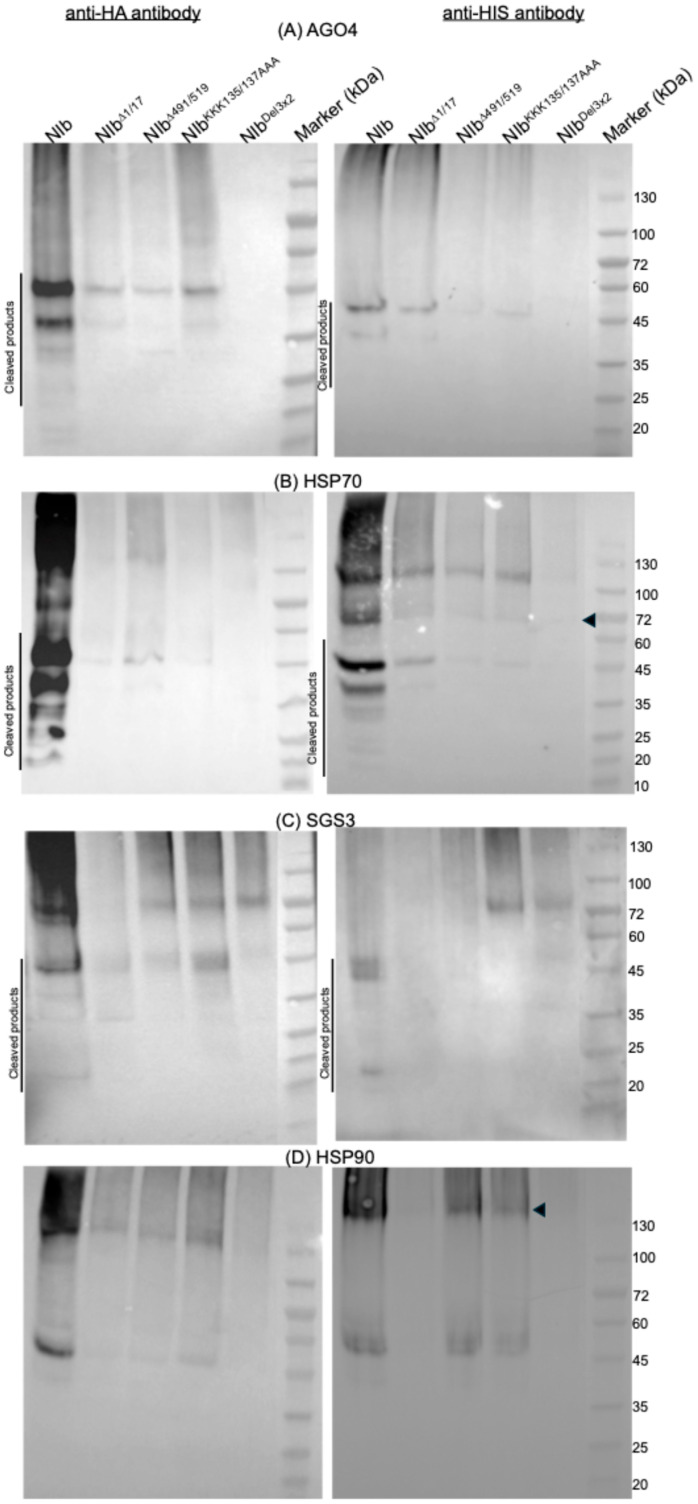
NIb mutants do not interact with AGO4, HSP70, or SGS3, as detected with anti-HA tag (left panel) and anti-HIS tag (right panel) antibodies. Analysis, under native conditions, of samples co-expressing NIb and AGO4, showed cleaved protein complexes. In contrast, no such complexes were found in samples when NIb^Δ1/17^, NIb^Δ491/519^, NIb^KKK135/137AAA^, or NIb^Del3×2^ was co-expressed with AGO4. Similar to (**A**) AGO4, co-expression of NIb constructs with (**B**) HSP70 (the arrowhead indicates a monomeric HSP70) and with (**C**) SGS3, respectively, showed no accumulation of protein complexes. No cleaved products were observed in co-expressions with (**D**) HSP90; the arrowhead indicates the HSP90/NIb mutant complex. The SDS-PAGE Coomassie blue stain of total protein lysate for Ni–NTA affinity purification can be found in Supplementary Figure S3. M: Novus Biologicals Blu11 Prestained Protein Ladder.

Assessment of NIb and mutants was carried out by co-expression with AGO4, HSP70, HSP90, and SGS3 in *N. benthamiana* plants and protein complexes isolated with Ni-NTA followed by Western blot analysis using HA, as described above. Results confirm interaction between NIb and AGO4, HSP70, and SGS3, respectively, leading to apparent degradation (Figure 11). In contrast, no protein complexes were observed in tissues co-infiltrated with mutants NIb^Δ1/17^, NIb^KKK135/137AAA^, NIb^Δ491/519^, and NIb^Del3×2^ indicating that interaction between NIb and AGO4, HSP70, and SGS3 likely plays a key role in NIb suppression of RNA silencing. Similar to wild-type NIb, NIb^Δ1/17^, NIb^KKK135/137AAA^, and NIb^Δ491/519^ were observed to interact with HSP90. These results therefore suggest that interaction between NIb and HSP90 is likely not the determinant in NIb suppression of RNA silencing. Given that NIb^Δ1/17^, NIb^KKK135/137AAA^, and NIb^Δ491/519^ are excluded from the nucleus (Figure 4), these results confirm that nuclear localization is likely required for NIb suppression of RNA silencing.

## 3. Discussion

Here, we demonstrate that PVY-encoded NIb suppresses host antiviral RNA silencing, yet this is only one of its several other actions against host immune responses. For example, TuMV NIb has been found to be sumoylated by small ubiquitin-like modifier 3 (SUMO3) and also to bind to SUMO-conjugating enzyme 1 (SCE1), thus making SUMO3 unavailable to activate the NPR1-mediated immune response to viral infection [12,18,47]. NPR1 is a master regulator of salicylic acid-mediated immunity. TuMV NIb sumoylation also reduces sumoylation of pelota, which is involved in the degradation of potyviral genomic RNA [48]. NIb suppression of antiviral RNA silencing, therefore, adds to the repertoire of NIb functions that counter host immunity.

This study shows that PVY NIb suppression of RNA silencing depends on nuclear localization, and we identified the ^135^KKK^137^ motif as a previously unreported NIb NLS. This motif is also found in the NIb of several potyviruses, including TuMV [49], tomato necrotic stunt virus [50], sorghum mosaic virus [51], and pepper yellow mosaic virus [52]. A search of the NIb NLS using an online NLS prediction tool [53] identified ^133^GGKKKD^138^ as the NIb NLS, which is in agreement with our results. Also playing a role in NIb suppression of RNA silencing is the C-terminal region, where the construct NIb^Δ491/519^, which lacks the C-terminal 28 amino acids, and the construct with a mutated acidic motif ^491^DEEE^494^ (NIb^DEEE491/494AAA^) exhibited significant loss of RNA silencing suppression capability.

Here, we found that PVY-encoded NIb interacts with at least four proteins in the RNA silencing pathway, namely, HSP70, HSP90, AGO4, and SGS3. Except for HSP90, these interactions lead to degradation of the interacting complexes. These proteins play key roles in host antiviral RNA silencing. For example, HSP70 recruits HSP90 to the RISC in *Drosophila* and human [45], as well as plant [54] systems. Mechanistically, in the nucleus, HSP70 binds and transiently pries open the otherwise closed AGO, and HSP90 is recruited into the complex, a process that stabilizes the open and active AGO, which then receives a sRNA duplex [55,56]. By interacting with HSP70/HSP90, NIb likely inhibits siRNA recruitment into the RISC, resulting in suppression of RNA silencing.

The failure of constructs with a mutated NLS to suppress RNA silencing can be explained by the fact that loading of siRNAs to AGO4 by HSP70/HSP90 occurs in the nucleus [45]. Additionally, although the SGS3/SDE5/RDR6 complex is formed in the cytoplasm where synthesis of ssRNA to dsRNA occurs, SGS3 primarily localizes in the nucleus [57,58]. Hence, interaction between NIb and SGS3 likely occurs in the nucleus and blocks SGS3 nuclear export to the cytoplasm to form the SGS3/SDE5/RDR6 complex. However, the fact that there were similar levels of siRNAs in silenced tissues and non-silenced tissues suggests that the interaction between NIb and SGS3 is likely not critical in NIb suppression of RNA silencing. Interestingly, the VPg of another potyvirus, turnip mosaic virus (TuMV), was previously reported to suppress RNA silencing by degrading SGS3 [38].

Canonically, AGO4 is known to associate with repeat-associated siRNAs (rasiRNAs) to control transcriptional gene silencing through methylation [59]. In this process, 23-nt/24-nt or 24-nt/24-nt siRNA duplexes are loaded into AGO4 in the cytoplasm, with the 24-nt siRNA strand acting as the guide that directs AGO4-siRNA complex entry into the nucleus, where the 24-nt siRNA strand binds to the cognate nascent RNA transcript at the locus where DNA methylation occurs [60,61]. Interestingly, AGO4 has also been found to interact with RNA silencing suppressors of several RNA genome viruses, including cucumber mosaic virus 2b [62], alfalfa dwarf virus P [63], and tobacco rattle virus 16K [64] proteins. Therefore, interaction between NIb and AGO4, leading to degradation of the complex, adds to the ability of plant RNA viruses to counter host defense strategies through interaction with AGO4, hence creating a favorable cellular environment for viral proliferation. This is a new role for AGO4, in addition to its well documented role of regulating gene expression through DNA methylation.

Interestingly, HSP70 also targets and degrades the potyviral CP to promote genome RNA replication [65,66]. Moreover, consistent with observations made in this study, HSP70 was reported to cause the degradation of turnip mosaic potyvirus RdRP [14]. It is important to note that NIb RdRP activity is regulated by Beclin1 (ATG6), which interacts with NIb, and the interaction complex is targeted for autophagic degradation [17]. How interaction between NIb and HSP70/HSP90—which leads to degradation of the NIb/HSP70 complex, as shown in this study—and involvement of these proteins in the VRC formation contributes to the overall virus infection process will need to be addressed.

## 4. Materials and Methods

### 4.1. Plasmid Construction and Agrobacterium Inoculation

NIb proteins investigated in this study were from PVY strains PVY^NTN^, PVY^O^, PVY^N:O^, and PVY^N-Wi^ found in North America [67]. RT-PCR amplification of each cistron was carried out using the primers listed in Table 1A. For each cistron, *ATGGCC*, coding for methionine and alanine, was added to the 5′ ends of the forward primer for translation initiation. Amplification products were introduced into the Gateway^TM^ donor vector, pDONR/Zeo (Invitrogen; Thermo Fisher Scientific, Waltham, MA, USA) obtaining Entry^TM^ clones. These Entry^TM^ clones were shuttled into Gateway^TM^ binary vectors pEarleygate100 for RNA silencing suppression analysis, and pEarleygate101 for subcellular localization and protein–protein interaction analysis. The binary plasmids obtained were introduced into *A. tumefaciens* strain GV3101 using the freeze–thaw method [68], and cultures were grown overnight at 30 °C for infiltration.

**Figure 12:**
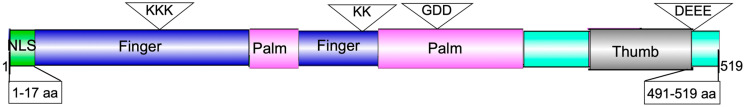
Representative diagram of the potyvirus NIb protein highlighting its functional domains. The conserved fingers, palm, and thumb subdomains of the RdRP are shown. Open inverted triangles mark amino acid residues that were substituted with alanine in this study, and open rectangles indicate truncations (deletions) introduced in NIb.

To identify amino acid residues involved in NIb suppression of RNA silencing, we introduced mutations in the NIb of PVY^NTN^, which is spreading increasingly in North America, while the incidence of PVY^O^, the ordinary strain, has been decreasing [69,70,71,72,73]. These mutations were introduced using the Q5 Site-Directed Mutagenesis Kit (New England Biolabs, Ipswich, MA, USA) with the primers listed in Table 1B. These mutations and NIb functional domains are illustrated in Figure 12. First, the N-terminal 17 amino acid residues that contain the recently reported NIb nuclear localization signal (NLS) [12] were deleted, obtaining the mutant designated NIb^Δ1/17^. A second truncation was produced by deleting the last 28 amino acids of PVY^NTN^ NIb, obtaining NIb^Δ491/519^. To identify additional NLSs, we searched for lysine-rich motifs, which are typically found in NLSs and identified motifs ^135^KKK^137^ and ^303^KK^304^. These lysine residues were replaced with alanine, obtaining NIb^KKK135/137AAA^ and NIb^KK303/304AA^, respectively. We investigated other conserved motifs, including the acidic motif ^491^DEEE^494^ and ^351^GDD^353^, which were substituted with alanine, obtaining NIb^DEEE491/494AAA^ and NIb^GDD351/353AAA^, respectively. The ^351^GDD^353^ motif is a critical component of the catalytic site within the viral RdRP protein [74]. An NIb null mutant, NIb^Del3×2^ (negative control), was generated from PVY^NTN^ NIb by deleting nucleotides GT at position bps 855–856 and truncating 50 bps from the 3′ end. These PVY^NTN^ NIb mutants were cloned into pEarleygate100 and pEarleygate101 binary vectors, as described above, and the recombinant plasmids were introduced into the *A. tumefaciens* strain GV3101.

### 4.2. Determination of NIb Suppression of RNA Silencing

Assessment of RNA silencing suppression was carried out using the GFP-transgenic *N. benthamiana* line 16c plants [41]. Plants were grown in a growth room with 16 h light and a temperature of 22 ± 3 °C. To induce *gfp* silencing in these experiments, we cloned the GFP ORF into pEarleygate100, obtaining 35S::GFP, which was introduced into *Agrobacterium* as described above. Overnight *Agrobacterium* cultures were pelleted and resuspended in an agroinfiltration buffer (10 mM MgCl_2_, 10 mM MES, pH 5.6) at an OD_60_ of 0.8. Cultures of each NIb construct were then mixed with those of 35s::GFP, and the mixture was co-infiltrated into *N. benthamiana* at the four-leaf stage using a 1 mL needleless syringe. GFP fluorescence was monitored using a handheld longwave UV lamp.

### 4.3. Northern Blot Analysis

To confirm that *gfp* is transcribed, we carried out Northern blot analysis of infiltrated leaves of *N. benthamiana* at 7 dpi. Total RNA was extracted using the TRIzol™ Reagent (Invitrogen; Thermo Fisher Scientific, Waltham, MA, USA), and DNA traces were removed with the RNase-free DNase I kit (Thermo Fisher Scientific, Waltham, MA, USA), as described by the manufacturer. The purity of the RNA was checked by a NanoDrop spectrophotometer (NanoDrop Technologies, Wilmington, DE, USA). Total RNA (20 μg) was heated at 55 °C for 5 min prior to separation in a 1.2% agarose/MOPS/formaldehyde gel in 1X MOPS buffer. The RNA was transferred overnight to a nylon membrane (Schleicher and Schuell, Keene, NH, USA) and hybridized with gene specific probes using the North2South Chemiluminescent Hybridization and Detection Kit, Inc. (Thermo Fisher Scientific, Waltham, MA, USA). Images were captured using a G:Box and GeneSys software version 1.8.11 (Syngene, Iselin, NJ, USA).

### 4.4. Determination of Subcellular Localization of NIb and NIb Mutants

For subcellular localization analysis, *Agrobacterium* cultures of NIb constructs were infiltrated into the abaxial surface of the first two true leaves of *N. benthamiana* and subcellular localization determined at 48 h post-infiltration. Here, infiltrated leaf pieces were excised, gently vacuum-infiltrated with water, placed on a slide, and covered with a coverslip for confocal imaging. Images were acquired on an upright Zeiss LSM 780 laser-scanning microscope (Carl Zeiss, Inc., Oberkochen, Germany) using a Zeiss 40X C-Apochromat (NA 1.2) objective lens. Multichannel images of yellow fluorescent protein (YFP) and chloroplast autofluorescence were acquired using the 488 nm line of an Argon/Krypton laser with 500 to 550 band-pass and 650 long-pass/390 emission filters, respectively. Images were captured as single optical sections or as a z-series of optical sections, and z-series data sets were displayed as single maximum intensity projection generated with Zeiss Zen Black software vSP2.

### 4.5. siRNA Analysis

siRNAs direct the RISC to target RNAs for degradation. In this study, we compared levels of small RNAs between *N*. *benthamiana* line 16c plant leaves infiltrated with NIb and those infiltrated with NIb^Del3×2^, the null mutant of NIb that does not suppress RNA silencing. For small RNA sequencing, samples were collected at 14 dpi from three biological replicates per treatment (n = 6 libraries total). Total RNA was extracted and libraries were prepared following standard plant sRNA workflows (5′ phosphate-dependent 5′ adapter ligation; 3′ adapter ligation; reverse transcription; PCR). Samples were sequenced as single-end reads long enough to fully cover 20–24 nt inserts. After sequencing, raw FASTQ reads were trimmed with Cutadapt v4.6 using error-tolerant 3′ adapter matching, and reads outside the retained small-RNA size range were discarded.

Trimmed reads were aligned end-to-end with no mismatches (Bowtie v1.3.1, -v 0) to GFP and NIb sequences, respectively. Alignments were sorted and indexed with SAMtools v1.22. To standardize coordinates and overlay sequence differences on depth tracks, we called variants directly from the sRNA alignments using SAMtools/BCFtools (samtools mpileup -Ou|bcftools call -mv, haploid). Sites used for figure overlays met depth ≥ 10 and QUAL ≥ 30. GFP and NIb, respective consensus FASTA sequences were built with the BCFtools software package (version 1.22) and used as targets for coverage and window-based tests. Per-base coverage was computed with deepTools v3.5.1 bamCoverage at binSize = 1 nt and normalized to counts per million (CPM). Strand-resolved profiles were produced from strand-split BAMs/bedGraphs (see scripts/make_strand_bedgraphs.sh). Group summaries show the library-wise mean (line) and 95% CI (ribbon).

Per-nucleotide coverage was computed in 1-nt bins and normalized as CPM (reads per million mapped reads). For aggregate comparisons, CPM was integrated along the transcript (cumulative running sum across nt; for non-1-nt bins, CPM was multiplied by bin width before summation) to obtain the transcript-wide siRNA burden. Group differences in per-library totals were evaluated by Wilcoxon rank-sum tests. Positional line plots show group means with 95% CIs; strand-resolved profiles were generated from strand-split alignments.

### 4.6. Identification of Host Proteins Interacting with NIb

To suppress RNA silencing, many silencing suppressors interact with proteins in the plant RNA silencing pathway. Here, to identify the host protein with which NIb likely interacts to suppress RNA silencing, we carried out an in planta interaction assay with nine genes in the RNA silencing pathway: AGO1, AGO4, DCL4, HSP70, HSP90, HYL, RDR6, SGS1, and SGS3. These genes were fused to 6×HIS tag in plasmid pYL436 [75], and each was co-expressed with HA tag-fused NIb constructs in *N. benthamiana* leaves. At 2 to 5 dpi, total protein was extracted from infiltrated tissues in a native extraction buffer (50 mM Tris, pH 7.4, 150 mM NaCl, 1 mM EDTA, 0.1% Nonidet P-40, and proteinase inhibitor mix), and centrifuged at 14,000 rpm for 25 min. Total plant protein (8 to 10 mg) was used to purify protein complexes by immobilized metal affinity chromatography (IMAC) with the Ni-NTA (nickel-nitrilotriacetic acid) magnetic bead kit (Thermo Fisher Scientific, Waltham, MA, USA) under native conditions. Ni–NTA agarose bead bound proteins were eluted and separated on ExpressPlus™ PAGE (4–20%) (GenScript, Piscataway, NJ, USA) native gel, followed by electroblotting onto a PVDF membrane. The membrane was incubated with a primary rabbit HA-tag antibody or HIS-tag antibody (Proteintech, Rosemont, IL, USA) at a dilution of 1:5000, and proteins were detected using HRP-conjugated goat anti-rabbit secondary IgG (H + L) (Thermo Fisher Scientific, Waltham, MA, USA), also at a dilution of 1:5000.

## 5. Conclusions

**Figure 13:**
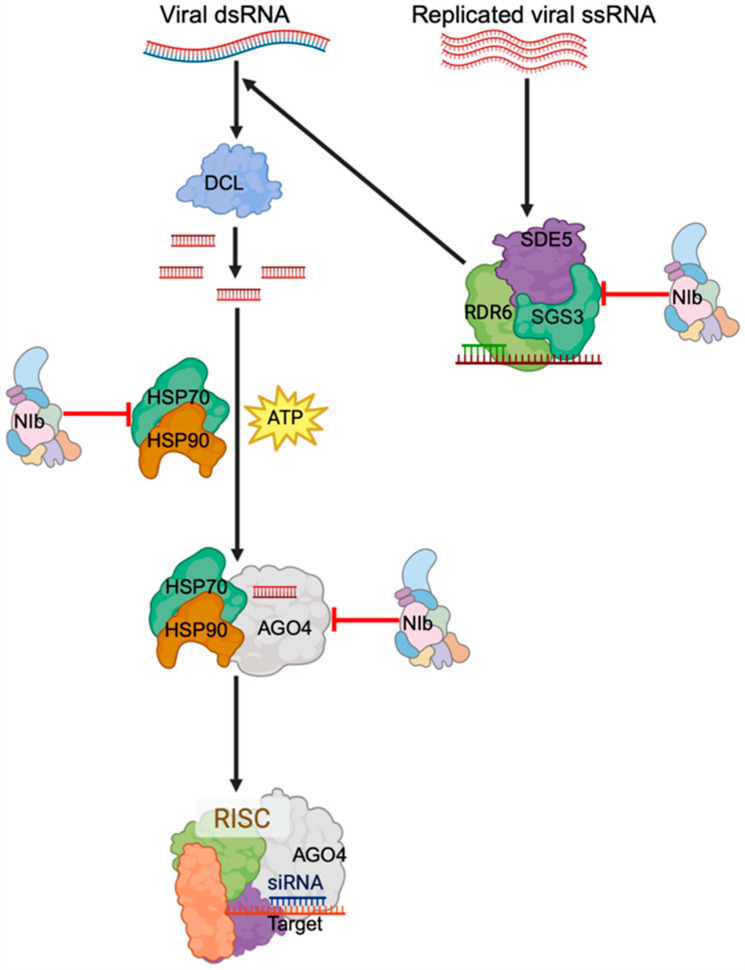
Antiviral silencing of RNA viruses. Viral dsRNA is processed into siRNA by DCL proteins. HSP70 then loads the siRNA into AGO proteins cooperatively with HSP90, which provides the energy needed, forming a mature RISC. The dsRNA is either from the replication intermediate or synthesized from the replicated genome by the RDR6/SGS3/SDE5 complex. This study shows that NIb counters this antiviral RNA silencing by targeting AGO4, HSP70/HSP90, and SGS3, respectively.

GFP silencing in GFP-transgenic plants is induced by 35S::GFP, where ssRNA GFP transcripts are converted to dsRNAs by RDR6 in cooperation with SGS3. The dsRNA is then processed by DCL proteins into double-stranded siRNAs, which are loaded into AGO proteins to form RISC [76] by HSP70/HSP90 in an energy-dependent process [46]. Once in AGO proteins, the double-stranded siRNAs are separated, and one strand guides the RISC to trigger global silencing of GFP transcripts in GFP-transgenic plants. Accordingly, antiviral silencing of RNA viruses is initiated when DCL proteins process viral dsRNA into siRNA. For (+)ssRNA genome viruses, including PVY, viral dsRNA is either a replication intermediate from annealed viral transcripts or it is synthesized from the replicated genome by RDR6 of the SGS3/SDE5/RDR6 complex. The dsRNA is then processed into siRNAs, which are loaded to AGO4 by HSP70/HSP90 to form a mature RISC. Given that similar levels of GFP-derived small RNAs were detected in both NIb and NIb^Del3×2^ treatments (Figure 5; Table S1), NIb suppression is unlikely to fully block siRNA biogenesis; rather, our data are most consistent with NIb acting downstream of siRNA production. The strong antisense bias of GFP-derived siRNAs in both treatments (Figure 6) indicates that GFP continues to be processed. From these observations, therefore, a model for NIb suppression of RNA silencing is proposed in Figure 13. Hence, NIb likely suppresses antiviral RNA silencing by targeting AGO4, HSP70/HSP90, and/or SGS3, leading to complex degradation. Hence, NIb interactions with AGO4 and HSP70 (and SGS3) are most parsimoniously interpreted as disrupting the use of these siRNAs in effector complexes, rather than blocking their biogenesis. Finally, NIb-derived siRNAs were observed to be more balanced between sense and antisense strands (Figure 8), consistent with their origin from a transiently expressed coding transcript, compared to a stably integrated GFP-transgene.

## Figures and Tables

**Table 1:** Primers used in characterizing potato virus Y NIb suppression of RNA silencing.

(A) Wild-Type NIb Primers		
ID	Sequence	Construct
NTN_NIb-F	ggggacaagtttgtacaaaaaagcaggcttaATGGCCGCTAAACATTCTGCG	
NTN_NIb-R	ggggaccactttgtacaagaaagctgggttTTGATGGTGCACTTCATAAGTATCG	PVY^NTN^
NIb. NO.Nwi.O-F	ggggacaagtttgtacaaaaaagcaggcttaATGGCCGCTAAGCACTCTG	
NIb.NO.Nwi.O-R	ggggaccactttgtacaagaaagctgggttTTGATGGTGTACTTCATAAGAGTCAAATTC	PVY^O^, PVY^N:O^, PVY^N-Wi^
**(B) Primers Used to Introduce Mutations**		
**ID**	**Sequence**	**Mutation**
NIb^ΔKKK^-F	AGCTGACTACTTCGAGCATTTTAC	NIb^KKK135/137AAA^
NIb^ΔKKK^-R	GCTGCGCCTCCATACATAGCTCC	
NIb^ΔKK^-F	AACAATTGTCGCTGCTTTTAGAGGTAATAATAGCGGTC	NIb^KK303/304AA^
NIb^ΔKK^-R	CCATCTGGAGTTGAGATTG	
NIb^ΔGDD^-F	TGCATTATTGATTGCTGTGAATCC	NIb^GDD351/353AAA^
NIb^ΔGDD^-R	GCAGCATTAACAAAGAATACACACG	
NIb^ΔDEEE^-F	GCAGCTCTGAAGGCTTTCACTGAAATG	NIb^DEEE491/494AAA^
NIb^ΔDEEE^-R	TGCAGCTACTGTCCTATTCATGTACAAC	
NIb^Δ1/17^-F	GTGGCGACAATGAAGAGTC	NIb^Δ1/17^
NIb^Δ1/17^-R	GGCCATTAAGCCTGCTTT	
NIb^Δ491/519^-F	AACCCAGCTTTCTTGTAC	NIb^Δ491/519^
NIb^Δ491/519^-R	TACTGTCCTATTCATGTAC	
NIb^ΔGT855–856^-F	ACACAGAAATAATTTACACAC	NIB^Del3×2^
NIb^ΔGT855–856^-R	AAATTGCGCAACATTTGC	

The lowercase sequences are attB1 and attB2 adaptor sequences for Gateway recombination. The ATGGCC sequence at the 5′ ends of the forward primers, coding for methionine and alanine, was added for translation initiation and to maintain the correct reading frame with the cistron.

## Data Availability

The raw data supporting the conclusions of this article are stored securely on the DNA and Data Science Core (dsDNA) server and will be made available by the authors upon request.

## Supplementary Materials

The following supporting information can be downloaded at https://www.mdpi.com/article/10.3390/ijms27031208/s1.
