# Supplementary material for: Potato Virus Y NIb Multifunctional Protein Suppresses Antiviral Defense by Interacting with Several Protein Components of the RNA Silencing Pathway
Source: Int J Mol Sci. 2026 Jan 25;27(3):1208. doi: 10.3390/ijms27031208 (PMC12898360; doi:10.3390/ijms27031208)
Supplement: Supplementary file 1 [file ijms-27-01208-s001.zip › ijms-4105122-supplementary figures.pdf]

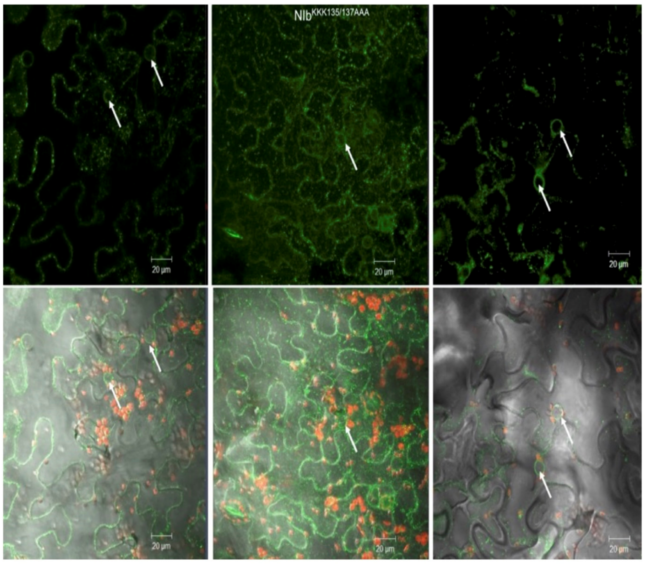

Figure S1. Confocal microscope images showing that $NIb^{KKK135/137AAA}$ is excluded from the nucleus (arrow), confirming that $^{135}KKK^{137}$ is a novel PVY NIb nuclear localization signal.

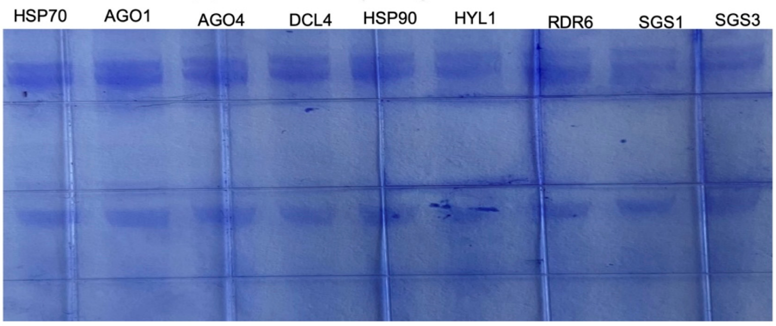

Figure S2. The SDS-PAGE Coomassie blue stain of the total protein lysate for Ni–NTA affinity purification in Figure 9.

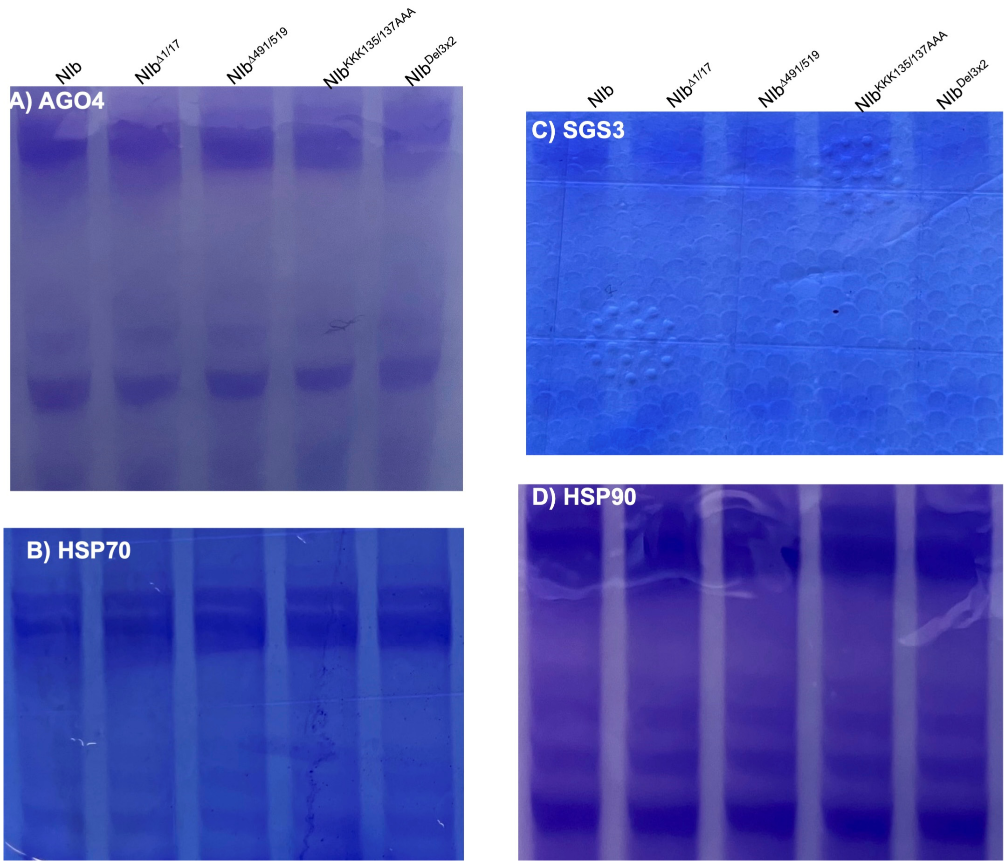

Figure S3. The SDS-PAGE Coomassie blue stain of the total protein lysate for Ni–NTA affinity purification in Figure 11.
