# Supplementary material for: Potato Virus Y NIb Multifunctional Protein Suppresses Antiviral Defense by Interacting with Several Protein Components of the RNA Silencing Pathway
Source: Int J Mol Sci. 2026 Jan 25;27(3):1208. doi: 10.3390/ijms27031208 (PMC12898360; doi:10.3390/ijms27031208)
Supplement: Supplementary file 1 [file ijms-27-01208-s001.zip › ijms-4105122-supplementary table.pdf]

Supplementary Table S1. Total GFP-mapping siRNA abundance (RPM) per library. Small-RNA libraries were prepared from GFP line 16c leaves co-infiltrated with 35S::GFP plus NIb or NIbDel3x2 at 14 dpi (three biological replicates per treatment; n = 6 libraries total). For each library, GFP-mapped reads were counted from GFP-alignment BAMs and normalized by the total number of adapter-trimmed small-RNA reads in the corresponding library (RPM = GFP-mapped reads / total small-RNA reads × 10^6). The percent of total small-RNA reads mapping to GFP is also shown.

Library ID	Treatment	Total small-RNA reads	GFP-mapped reads	GFP siRNAs (RPM)	GFP-mapped (% of small-RNA reads)
Nib_1_S10_L002_R1_001	NIb	7,363,559	9,560	1,298.3	0.1298%
Nib_2_S11_L002_R1_001	NIb	7,655,224	5,948	777.0	0.0777%
Nib_3_S12_L002_R1_001	NIb	9,086,681	13,489	1,484.5	0.1484%
Del2x3_1_S7_L002_R1_001	NIb^{Del3x2}	4,895,378	23,235	4,746.3	0.4746%
Del2x3_2_S8_L002_R1_001	NIb^{Del3x2}	8,994,944	40,721	4,527.1	0.4527%
Del2x3_3_S9_L002_R1_001	NIb^{Del3x2}	8,021,301	13,245	1,651.2	0.1651%
